# Growing up in transit. Personal development and resistance of migrant adolescents travelling through Mexico unaccompanied

**DOI:** 10.1016/j.jmh.2024.100245

**Published:** 2024-07-14

**Authors:** Susanna Corona Maioli, Delan Devakumar, Shoshana Berenzon Gorn, Rochelle A. Burgess

**Affiliations:** a UCL Institute for Global Health, UK; b Instituto Nacional de Psiquiatría Ramón de la Fuente Muñiz, Mexico

**Keywords:** Transit migration, Adolescent health, Psychosocial wellbeing, Violence, Identity, Resilience

## Abstract

Mexico is a major global migration corridor. The last decade has seen an increase in adolescents travelling unaccompanied from Guatemala, Honduras and El Salvador, mainly due to violence and natural disasters. This group of migrants is exposed to migration risks such as trafficking and exploitation, as well as multiple deportations and long waiting times without appropriate access to health and education services. These conditions have significant documented adverse impacts on health and mental health, but the impact on personal development has been less explored. Thus, this article aims to explore the impact of migration transit on identity and resilience of unaccompanied migrant youth, psychosocial mental health constructs that are important for wellbeing and personal development. Through 47 semi-structured interviews conducted with migrant youth and different migration workers in summer 2021 in two migrant shelters in Mexico City and Guadalajara, we found that possibilities for identity building for migrant youth were restricted in a context of curbed freedom. Violence and poverty led to loss of crucial formative years, pushing many to aspire only to work – regardless of the type of work. Resilience overlapped with resistance but was generally maintained with cultural narratives of goal achievement, faith and hope. Strong responsibility towards sending remittances to family made family one of the main goals of the migration journey. Workers highlighted how regional migration management, underlined by discriminatory policies which place profit and national security ideology before a recognition of common humanity, is a considerable barrier to funding services and advancing access to rights for youth. However, youth showed recognition of their own agency and humanity, which represents an important basis on which to build a counter-discriminatory vision and management of this migration. Results are reported according to COREQ guidelines.

## Highlights

• Lived experiences of migrant youth show maintenance of a positive, forward-looking projection of themselves related to their migration.
• The circumstances in which migrant youth migrate in Mexico are dangerous and precarious, blocking many avenues youth can have to access stable formative opportunities.
• Recognition of migrant youth's agency and interaction with peers are two fundamental ways to foster their resilience.

## 1 Introduction

Mexico is one of the main global migration corridors (Cárdenas Salgado et al., 2022). Currently, it is a country of transit or destination for people from more than 70 countries - mainly from Central and South America and Africa (Cierre de estadísticas diciembre 2021-enero 2022). Many crises are overlapping to promote this unprecedented increase in migration by land (Santos, 2023), although Mexico has been migrant territory for more than a century. Historical and intergenerational migration forms networks in the US that support future migrants (Massey et al., 1990). Migrants’ need to send remittances has been historically manipulated as an exploitable workforce, if undocumented and deportable (Akers Chacón and Davis, 2018; De Genova, 2002). In the last decade, there has been an increase in families, women and underage migrants travelling without legal guardians. Border ‘encounters’ by US Customs and Border Patrol with unaccompanied children have risen by more than 600% since 2012 (U.S. Customs and Border Protection 2024). Systematic global data on this group of migrants, termed ‘unaccompanied minors’^1^ is severely lacking (Corona Maioli et al., 2021) due to varying definitions applied; avoidance of detection; or inaccurate age assessments (Netz, 2022). Data breakdown by gender diversity or disability is essentially non-existent (Corona Maioli et al., 2021). Undocumented migration status leads to invisibility, in response to regional migration policies which favour ‘containment’ of migration. Increasingly difficult access to legal migratory pathways leads to finding hidden-away pathways: dangerous and controlled by organised crime (de la and Rodríguez, 2022). For unaccompanied children and adolescents, the risk of being kidnapped, trafficked or exploited is exacerbated (Comisión Nacional de los Derechos Humanos 2009; Amnesty International 2021). Many already come from situations of adversity - such as physical or sexual violence, negligence or poverty - and find that Mexico has no appropriate publicly funded spaces to protect them (UNICEF México 2019; Xantomila, 2022). Such experiences are associated with health and mental health challenges: nutritional deficiencies; poor dental health; drug abuse; post-traumatic stress disorder (PTSD), depression and anxiety (Corona Maioli et al., 2021) - which in turn impact adult health^2^ and wellbeing (Felitti, 2002).

Many health conditions of migrant youth are due to social adversities: complex and modifiable risk factors (Allen et al., 2014; Chase et al., 2019). While conditions such as PTSD or disabling injury at an early age must be taken seriously, we should avoid the assumption that migration always leads to traumatic outcomes. Thus, in this context, we aimed to explore the impact of migration transit on psychosocial aspects of migrant youth mental health – specifically identity and resilience – which acquire relevance regarding personal development and wellbeing.

### 1.1 Importance of psychosocial aspects of mental health

Psychosocial aspects of mental health are the interaction between individual psychological processes, and social relational processes - family, community, culture, politics – which become particularly important in contexts of social adversity (Agic et al., 2019; Derluyn and Broekaert, 2008). Social adversities – including violence and deprivation - are understood differently depending on their frequency and support available (Patton et al., 2016; Barber, 2008; Carlson et al., 2012).

We draw on two perspectives of identity to understand young people's migratory experiences: Glynis Breakwell's Identity Process Theory (Breakwell, 2015) and Gilberto Giménez's cultural identity theories (Giménez, 2018; Giménez Montiel, 2010). Both scholars highlight the interaction of identity with the social environment, often marked by discrimination in a forced migration context. Discrimination is based on identifiers which justify a specific ‘place’ in society, as favourable for some as it can be asphyxiating for others (Selvarajah et al., 2022). Continuity, an important pillar of identity (Jaspal and Breakwell, 2014), is challenged or lost during migration processes, especially if sudden and forced. Loss of stable social and spatial references can be more arduous for youth who have parents far away or completely absent, as previously described by Oppedal and Idsoe (Oppedal and Idsoe, 2015). Thus, identity building – especially during adolescence – is particularly demanding for this group of migrants. This makes the case for the importance of resilience for identity building in this context. Resilience, defined as ‘the capacity to recover from difficulties’ (Luthar et al., 2000; Southwick et al., 2014; Fergus and Zimmerman, 2005), becomes paramount for withstanding both normal migration related difficulties (acculturation, uncertainty, loss) and traumatic or violent experiences related to undocumented migration. Despite traumatic experiences, instances of good functioning have been documented to outnumber pathologic outcomes (Panter-Brick and Eggerman, 2011; Jones, 2008). Thus, both identity and resilience are dynamic psychosocial processes which interact with the social environment and determine personal development and wellbeing. For example, a prior review outlined the beneficial effect that social support and access to health and education can have for mental health and development of migrant youth (Corona Maioli et al., 2021). However, most studies focus on post-settlement experiences, and the role of migration transit as a social environment is less clear.

### 1.2 Migration transit as a social context

A transit state is intuitively considered a passage on the migration route, situated geographically between an origin and a destination. We conceptualise migration transit beyond a geographical space: a socially created space, dependent on geopolitical dynamics and direction of migratory flows (Collyer et al., 2012; Hess, 2012). It is a social context, complicated by the misleading temporariness of the word ‘transit’ that assumes non-permanence. In fact, transit states are increasingly destinations due to the practice of border ‘offshoring’ (Missbach and Phillips, 2020): represented by agreements such as those between the EU and Tunisia (ec.europa 2023; The IRC 2023), or USA and Guatemala (Pushing Back Protection 2021). Natural barriers like the Darién jungle, the Sahara or the Sonoran desert, and the Mediterranean sea become part of outsourced migration management, by being sites where migrant deaths occur with little government accountability (Schindel, 2020).

Mobility represents a redistribution of access to resources, painstakingly monitored by governments through selective access (De Genova, 2002). However, as argued by Sabine Hess, the social context of migration transit is inevitably fluid: people stay where they have work, family or a community, sometimes regardless of their categorisation as ‘asylum seeker’, ‘transit migrant’ or ‘refugee’ (Hess, 2012). Endless pathways to regularise migration status result in people finding a way to exist throughout the “series of stops, starts, reverses and circularities” of undocumented migration – as stated by Sue Clayton (pp 115; (Clayton and Gupta, 2019)). Travel or waiting can last years, with descriptions of life ‘in transit’ as an existential limbo, a quest for a future projection, but also a state of hope and resistance (Erazo, 2019; Sampson et al., 2016; Ghorashi et al., 2018). For migrant adolescents, with or without physical parental presence, this experience is likely to heavily impact transition to adulthood (Oppedal et al., 2017). For example, being segregated in a camp curbs opportunities which may be the source of hope (Jones, 2008). In Mexico, the authorities which have a mandate to protect children – including migrant children –are the *Desarrollo Integral de la Familia* (DIF) or child welfare and the *Procuraduría de Protección a la Infancia* or Child Protection Authority, according to a law established in 2014 called the *Ley General de los Derechos de Niñas, Niños y Adolescentes*. However, a lot of the work falls on civil society shelters.

## 2 Material and methods

In the context of a motivated ethnography, which enquires a social context with a specific focus (Burgess, 2016), qualitative semi-structured interviews were conducted with a sample of 18 unaccompanied migrant youth aged 14 to 19, and 29 professionals in services for migrant youth (table 1). The sample of migrant youth was selected to represent transition to adulthood as a process: child protection must include a continuity for the post-18 year cutoff (Save The Children 2016).

**Table 1:** Professions of worker participants.

Role	Participants
Shelter psychologist/psychiatrist	3
Shelter lawyers	2
Migration academics	3
Shelter or NGO coordinators	5
Shelter volunteers	2
Shelter sister	1
Shelter staff	1
Shelter directors	4
Child Protection Authority (Procuraduría) Officers	2
Shelter or NGO social workers	2
UN workers	2
INM Child Protection Officer	1
UN Child Protection Officer	1

Fieldwork took place in June and July 2021 in two migrant shelters in Mexico City and Guadalajara. As most shelters in Mexico, these spaces were founded by religious organisations and functioned as civil society (not government-run), based mostly on private and international donations. Fieldwork concluded prematurely because of the Covid-19 pandemic. Prior to data collection, SCM conducted two months of volunteering in the shelters, in order to scope fieldwork feasibility and obtain familiarity with shelter staff. Ethical approvals were obtained from the Ethics Boards of Instituto Nacional de Psiquiatría (IRB600006105) and University College London (16,797/001). Because research activities were conducted within the activities offered by the shelter, there was no compensation for travel or other monetary payment. Migrant youth were given a leaflet with their rights in Mexico and institutions to contact for help, a sticker, drawing album and charcoal pencil as gratitude for their time.

### 2.1 Recruitment

Unaccompanied migrant youth were recruited from the two shelters by convenience sampling. A first session was organised as an introductory explanation of the research and ethical processes. A week later, drawing workshops were organised with participants in groups of 5 to 10. A week after, an in-person interview was organised individually. All provided oral and written consent and were anonymised with a pseudonym. Professionals were recruited by email, at the shelters and by snowball sampling.

### 2.2 Drawing workshops and interviews

Migrant participants were asked to represent three concepts in drawing: *tránsito* (transit, explained as ‘moving from one place to another’)*, identidad* (identity, explained as ‘how we see ourselves and are seen’) and *resiliencia* (resilience, explained as ‘what gives us strength’). These drawings were used during subsequent interviews to include a visual elicitation approach, which involves introducing images in an interview to evoke more or different information than would words alone. This method enables representing feelings or abstract concepts with symbols, which may be an easier expression for young people (Moskal, 2017). An additional 10 pre-prepared images^3^ were also used. Interviews with migrant youth covered basic demographic information (table 2) and then followed a topic guide (annexed). Interviews with workers regarded their opinions on the social context faced by migrant youth in Mexico. All interviews were conducted in Spanish, recorded and transcribed.

**Table 2:** Demographics and social history of migrant youth participants.

Demographic indicators	Number of participants
**Age**	
14	4
15	2
16	1
17	4
18	6
19	1
**Gender**	
Boy	12
Girl	4
Transgender girl	1
Other	1
**Nationality**	
Honduras	7
Guatemala	6
El Salvador	4
Mexico	1
**Main reason for leaving**	
Gang violence / threats	6
Poverty	5
Family violence	4
Family reunification	2
Discrimination	1
**Time since left country**	
< 3 months	5
> 6 months	13
**Migration status**	
(Waiting for) family reunification	5
Asylum seeker	4
Refugee	4
Humanitarian visa	3
Planned return	2
**Time in city of interview**	
< 3 months	7
3–6 months	4
> 6 months	7
**Accompanied by**	
Non-guardian family	6
Smuggler	4
Nobody	4
People known on the way	2
Friends (previously met)	2
**Support in country of origin**	
Yes	13
No	5
**Support in country of destination**	
Yes	10
No	8
**Level of education (completed or uncompleted)**	
Primary	14
Secondary	4

### 2.3 Data analysis

Reflexive thematic analysis (Braun and Clarke, 2006) was conducted in Spanish, later translated in English. After familiarisation with the data (re-listening to interviews and transcription), codes were identified and organised into subthemes and themes using NVivo 12 software. Themes were further organised into thematic categories, allowing clear argumentative claims about the data (Burgess et al., 2022). Coding was conducted both inductively and deductively, verified by a senior author who is a qualitative research expert. Based on the research focus and a social constructivist epistemological basis, a deductive process took place by keeping the interaction between identity, transit and resilience as theoretical lens of analysis. Simultaneously, flexibility was maintained in an inductive approach. Analysis was conducted for migrant youth and workers as two separate datasets, but there was intentional code overlap to allow different perspectives of the same code when relevant. Validation was conducted by triangulating the independent coding of 20 interviews by two research assistants. Analysis was considered complete at code saturation, when no new codes could be generated. We note that this reflects saturation of our subjective interpretations of the data, rather than complete theoretical saturation of meaning (Braun and Clarke, 2021).

### 2.4 Reflexivity

Reflexivity of the first author, with direct involvement in data collection, is that of a young woman, Mexican and Italian by nationality. SCM speaks both languages fluently, identifies as a cultural mix and is supportive of fair movement of people. Regarding power imbalance given by easier mobility, or by ‘adult’ age, SCM strived to maintain a horizontal attitude in the field: especially important in migration contexts, where giving the ‘right’ answers is often required for migration interviews (Chase et al., 2021). All other authors did not have contact with participants. DD is a UK-based academic and migrant, who moved to the UK as a child and whose family's history is that of forced migration, fleeing conflict. SBG is a happy migrant who migrated from Costa Rica to Mexico and is sensitive to the needs of migrant population. RAB supervised the qualitative analysis, she is a UK-based scholar-activist and migrant of Black Caribbean heritage, with lived experience of mental health conditions and violence.

## 3 Results

Analysis identified eight thematic categories (annexed), of which five are discussed here following the concepts of relevance to this article: migration transit; impact on identity; resilience and resistance. Two thematic categories pertaining to the role of professionals in safeguarding unaccompanied youth will be explored elsewhere (forthcoming). Findings related to the final thematic category are also reported elsewhere (Corona-Maioli et al., 2024).

### 3.1 Migration transit

This section presents the circumstances unaccompanied migrant youth experienced in their places of origin and during their journeys. Two thematic categories are explored: *embedded violence in the environment,* and *vulnerabilities of migrant youth from worker perspective.*

#### 3.1.1 Embedded violence in the environment

Participants confirmed the increasingly violent context that has been documented in Central American countries (Sawyer and Márquez, 2017; UNHCR 2021). No participant attempted going to the police before migrating: possibly due to an expectation of impunity and corruption (Kauth, 2000), fear that threats extend to other family members, or to violence stemming from family itself. Three types of violence were identified in interviews, often overlapping: gender-based, gang, and family violence.

Gender-based violence was experienced in the form of discrimination, sexual abuse and attempted forced marriage. Violeta, an 18-year-old transgender participant, left her family home because of gender-based discrimination. She describes violent experiences occurring to her and other gay friends in Honduras:

V: *“We grew up together, them as gay guys and I as a trans girl. But it got to an extreme of… honestly, they treated us very bad. They killed two of them, the rest left. I was the only one that remained. I escaped them killing me, thank God nothing happened but they were about to, they had me in their hands”*

S: *“Did they do anything to you?”*

V: *“No, but they did threaten me with a knife and a gun”*

Violeta joined a gang for six months when she left her family home, at 11 years of age, because in the gang she “*felt a bit more free, away from* [her] *family's discrimination and maltreatment*”. In the gang, she experienced sexual abuse and was forced to perpetuate violence. She recalled how gang members beat her because she did not ‘finish a job’:

S: *“If you want to tell, what was your role with* [the gang]*?”*

V: *“Well, ehm, stealing, I never stole, honestly, they did. I kept watch, making sure police wasn't around. If police or* huachos, *as we call them, came, I made signs or flied a* mara *flag and everyone understood and left. And killing. They forced me to kill* [a lady] *but, honestly, after I did it I felt uncomfortable* (…) [And] *they finished her because it made me sad to see how she was moaning and I did not have the courage to keep killing her. And because of that they beat me, because I did not want to finish the job.*”

Erica, 14, from Guatemala, had to leave her country because her father, who was a gang boss, tried to forcefully marry her. After she left, her father sent her death threats by message. She described how he also used to beat her friends, possibly as a way to protect himself against potential enemies:

E: *“Every time I had a friend he got angry and sometimes he hit me, or he ordered my friends to be hit, so they would go away”*

S: “*Why did he want you to be isolated*?”

E: “*I don't know, he always wanted me locked up* (…) *because he was a drug dealer my friends did not want to be with me.*”

Thus, gang violence intersects with gender-based and family violence. Other participants also experienced threats for refusal to join a gang. Estrella, from Guatemala, who had to leave the family home at 13 due to discrimination over sexual preference, was working and living alone in Guatemala City and had to quickly migrate to Mexico because she was beaten by gangs after her refusal to join. She also recalls a very lucid decision of not wanting to join, when she was just 15:

E: *“They offered me good payment, but I was going to last one year alive. Or less. So that didn't have a future, why should I get into something that I know doesn't end well”*

(…)

S: “*Did you look for a coyote* [to migrate]*?”*

E: *“I didn't have time* [to look] *for a coyote, no. I simply said… they beat me. About three weeks before I left the country* [the gangs] *beat me really bad… because I didn't accept* [to join], *right?* (…) *They broke my nose, my lips…”*

S: “*They stalked you outside your job?”*

E: “*I was going home and then… there was a very dark street* (…) *So in that street they took advantage and…* [then] *people came to help so they ran.*”

Family violence was experienced by five participants as neglect, physical and emotional abuse, and, in the case of Honduran brothers Héctor and Gustavo, death threats from a cousin. Héctor recalls how the problem with their cousin started out of a common interest over a girl, but the cousin “*had guns, he had been two years in jail for underage people, because he had killed two people one with a machete and another one with a gun*”. This story exemplifies how the culture of violence can spread beyond gangs, and how incarceration can have a backfire effect:

S: “*Why do you think your cousin is like that?”*

H: “*I don't know, for sure he is not afraid to go to jail* (…) *That's what he told us, that he had been in jail once and he was not afraid to go back. He did not care to kill a person, if he knew what jail was like”*

Violence then continues on the road, for example on the dangerous cargo train of La Bestia that migrants climb on to get to the Northern Mexican border. Alex, a 13-year-old participant from Guatemala, shares:

A: “*I saw how the train mutilated a girl, an adolescent, of 15–16 years* (…) *I fell sometimes from the train, but they were mild falls.* [Still], *how could I not be afraid* [of sleeping along train tracks], *if I was risking my life*”

**Fig. 1:**
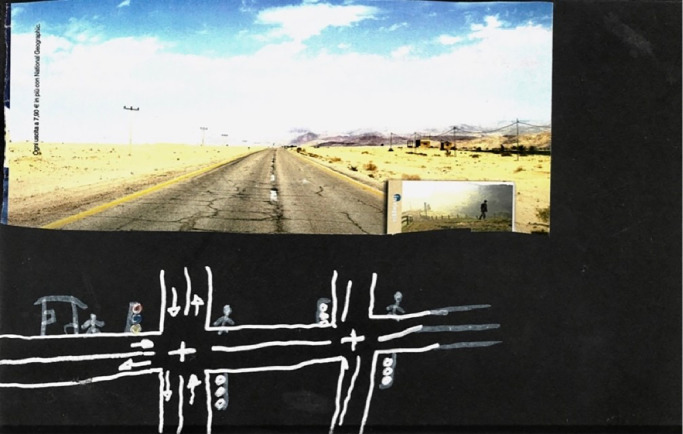
Manuel's drawing, representing transit.

Manuel, an Honduran 18-year-old participant, lost his left leg by falling off the train. In his drawing (Fig. 1), he represented his journey and reflected that “*maybe it would have been better never lo leave my country*”. Still, at the time of the interview he was planning to stay in Mexico and find a job to support his family.

**Fig. 2:**
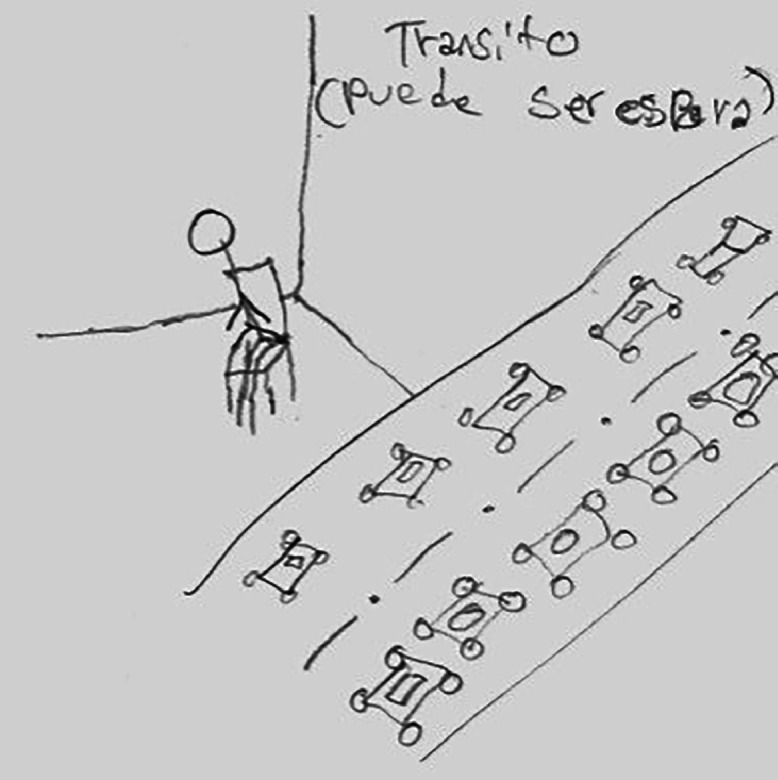
Section of Romeo's drawing representing transit as waiting. Translation from the drawing: *transit (can be waiting)*.

The direct violence many youth experienced intersects with structural inequalities and discrimination, which curb the freedom required to work or study peacefully. Being in transit means disrupting stability and continuity, identified for example in participants who had migrated multiple times. However, the main disruption to stability is perhaps the long waiting times of a regularisation process. Namely, obtaining refugee status or approval for family reunification implied waiting in the shelter, without a certain future projection. This is represented in Romeo's drawing (Fig. 2), a 14-year-old participant from El Salvador, who drew ‘transit’ as *la espera*, or waiting.

Long waiting times lead to despair and escapes from institutional safeguarding, as reported by two participants. Alex mentioned how being in institutional care limited making one feel at home:

“*Sometimes I feel like* [the shelter] *is my home, but other times I don't. Because there are people that come and go* [and] *others that talk behind your back and make you feel like a stranger*”

Migrating with little possibilities to envision a future becomes thus a project that is normalised as difficult. Rosendo, 17, from El Salvador, shares:

R: *“There where I'm from, many cross* [the border with the US] (…) *Just one cousin of mine did 5 trips and the fifth one he crossed. Yes, they always deported him*”

S: “*Mhj. Are you afraid of deportation?*”

R: “*Mmmm. Yes, because I'd have to start my trip* [again] *from scratch basically*”

#### 3.1.2 Vulnerabilities of migrant youth from professional perspective

Workers identified three main risks unaccompanied migrant youth were exposed to in relation to migration transit in Mexico: trafficking, exploitation and substance abuse. These risks can be considered as, at the same time, contributing to and originating from the violence-permeated contexts described above (Kiss et al., 2015). This is mentioned by a Procuraduría officer:

*“As they find no way out of violent circles, these kids are very sought after by organised crime”*

Trafficking^4^ situations described by workers included sexual exploitation (mostly of girls), working as *halcones*,^5^ and Mexican children at the border smuggling drugs or people, coerced through a “*payment that would be very difficult to get in Mexico*” (shelter director). Indeed, having few legal work options can push towards trafficking networks, while substance abuse may be a response to the psychological cost of daily survival (Cardoso, 2018) or violence:

Shelter director: “[We see] *not only emotional harm, but also very delicate psychiatric cases because of those life experiences of violence received, exerted and accumulated”*

Shelter psychiatrist: “*Migration should not necessarily be adverse for mental health, but it is the conditions in which* [unaccompanied youth] *migrate, unprotected, which obviously will increase the risk* [of substance abuse]*, especially if there is predisposition* [to it]”

School interruption was also emphasised as playing a role in mental health vulnerability, because literacy provides tools to understand the world and oneself:

Shelter psychiatrist: “[Some migrant youth] *may not know how to read or write, which has a lot to do with health* (…) *because everything adds up when there are adverse psychosocial circumstances* (…) *which can result in a* [mental health] *pathology*”

Shelter psychologist: “*I think some of the clinical implications* [we see] *have to do with the difficulty adolescents have to express verbally their distress*”

There was no mention of these vulnerabilities among migrant youth themselves.

### 3.2 Impact of migration transit on identity

This section describes the impact on identity development of the circumstances unaccompanied migrant youth experienced, exploring two thematic categories: *lack of freedom to develop and choose*, and *politics and vision.*

#### 3.2.1 Lack of freedom to develop and choose

This thematic category captures how lived experiences of migrant youth interviewed reflected a restrictive environment, often barring formative opportunities. Direct and structural violence, outlined in the section above, are clear examples of the restrictions these youth experience. For example, Estrella shares how she felt she had no choice but to migrate after the *maras* beat her: she sold everything of value she had and left. Often, family negligence and maltreatment create the situations of risk that youth are exposed to, who end up leaving school, in institutional care or experiencing multiple migration journeys.

Lack of a stable future projection may hinder building a stable identity, because stability is required in a formative process. For migrants, stability begins with migration regularisation. Three participants, William, Jose and Manuel, all 18 and from Honduras, shared how important it was for them to receive legal counselling in shelters. Thanks to this regularisation, William, who had no family support, was able to project his future in Mexico envisioning a career in boxing:

W: “*I have a goal and that is to become a professional boxer* [and] *study business so I can open my own gyms and administer them* (…) *I want to open gyms for people who do not have a lot of money. But all in its own time*”

Similarly, Violeta, who had also obtained refugee status, shared the difficulties she encountered at obtaining a job due to discrimination for her gender identity, but she was working at the time of the interview and had a firm position on not giving up on this identity:

V: *“When someone treats me badly, sometimes I gather more strength because I say, not because this person told me I'm useless will I feel low. No, I will move forward, I will fight because I know who I am and what I want, and I will fight for it.*”

Interestingly, these participants who showed very marked personal plans had no family support and so could envision their choices for themselves, as opposed to having the plan to send remittances which was encountered in many other participants, such as Alberto, 16, from Honduras. Envisioning a future project related to the family may help to alleviate the loss:

A: *“At first it cost me a bit because I was not used to being separated from my family, and I didn't know when I would see them again* (…) *With time I got used to it and managed to control how I felt. And now sometimes I get depressed, but I try to not think about it, to not demotivate myself”*

S: *“Have you given a reason to this journey?”*

A: “*Well, since I had the opportunity to travel, I've tried to help my dad, mainly. Because in my country* [Honduras]*, he is a taxi driver, and things cost too much over there* (…) *So in the US I plan to work and study, but with the main reason to help my dad. So he doesn't suffer too much”*

Although family has a clear protective role, it can also be restrictive in terms of identity building in the context of a restricted environment which allows few work opportunities for youth (Corona-Maioli et al., 2024). In other words, youth who had the goal to send remittances seemed much less focused on the type of work they would *like*, as expressed by this volunteer staff at a migrant shelter:

“*Most of them have no intention of personal educational growth, but they do have the aspiration that their parents or family in country of origin can benefit from anything they could do”*

Despite the restrictiveness of violence or of few work opportunities, the migration journey was often shared as a learning opportunity and a stimulus for personal development. For example, when participants were asked how the journey had changed them, varied responses were given emphasising how different individual trajectories are lost to generalised conceptualisations of migration:

Jose, 18, from Honduras: “*I have changed my way of thinking since I left my house, since I started my journey, I started maturing*”

Sara, 15, from Guatemala: “[This journey] *was a completely positive change* (…) *I was very impulsive, always in a bad mood… not anymore. I understood there is only one life and you have to laugh and live it”*

Alex, 15, from Guatemala: “*I didn't manage what I wanted* [to reach the US] *but I'm on something else… I'm learning different things”*

#### 3.2.2 Politics and vision

Political and social vision of migration play a key role in allowing settling in a different place, enabling the future projection outlined above as crucial for identity building. Currently, restrictive migration politics and social discrimination create barriers for migrant youth to access a continuous formative process (school, vocational opportunities or stable work). This thematic category captured, in professionals’ opinions, how the criminalising political vision of migration facilitates social discrimination of migrants, partly through the underfunding of appropriate services oriented to migrant youth protection or integration:

Shelter coordinator: *“There is an important limitation in terms of resources* [for services for migrant youth] (…) *We can think of local strategies, amplify our networks, keep on insisting* (…) *But unfortunately, what could mean a turning point for child protection depends* [more] *on authorities’ will and the resources assigned to this”*

Institutional and legal frameworks are technically in place but are not functioning appropriately, which comes down to a matter of lack of political will, or political will oriented to militarisation, as mentioned also by this shelter director:

*“In my experience the government has been the worst obstacle, the worst difficulty we have had to deal with constantly. For some time, this has to do with the messages that the government has sent through the media, of seeing migrants like a threat, like an enemy, like a criminal that has to be persecuted”*

A migration academic notes how securitisation can easily overlap with cruelty and social normalisation of migrant discrimination, most evident in the ‘Trump era’:

“*I think what we witnessed between the Trump era and the present is that the Trump era brought us to normalise an open practice of torture and cruelty towards migrant children that we had not seen before.*”

In fact, policies such as *zero-tolerance*^6^ left a dangerous precedent of attacking the most intimate sphere of a person, the family, for the ‘punishment, discouragement and control’ of undocumented migrants (Glockner, 2020), including small children who arguably need their families for survival. A UN Child Protection Officer described how another policy, the Migrant Protection Protocols (MPP),^7^ also created family separations:

“[Migrant families] *were waiting for their asylum hearings in the northern Mexican border,* [they] *were desperate. With no water, no food, violence, etc. So* [the parents] *thought, since the kids could not be returned under MPP, if we are going to die here* [in Mexico], *then better that our son survives. And they sent him alone to the desert, so he could be captured by the Border Patrol and sent to an ORR* [Office of Refugee Resettlement]”

A migration academic highlights how opposing migration is in line with maintaining an oppressive system based on reinforcing nationality as the main political identity, and neoliberalism as the main political and economic ideology:

“*Irregularised migration is possibly the most mediatised and politicised* [because] *it irrupts into national order and it is a form of rebellion against a terribly oppressive and unequal system which is modern neoliberalism*”

This unequal system impacts personal development and identity building, subordinated to passport type and level of wealth: the less powerful, the less wide-ranging are the options. Workers also highlighted how social acceptance of migrants, including those who have a legal permit to work, depends highly on public representation and adequate information about migrant rights to employers, civil registry officers, healthcare workers, educators, etc.

### 3.3 Resilience and resistance

This section presents the resources which represented resilience and resistance for migrant youth, as identified in interviews. The thematic category explored is ‘salir adelante’ *as a narrative of hope, personal development and goal achievement*’.

**Fig. 3:**
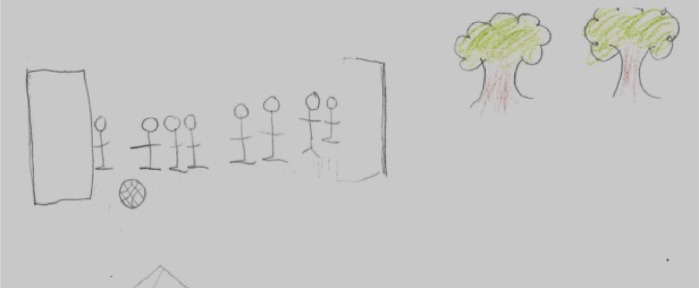
Section of Pedro's drawing representing a soccer game as resilience.

Migrant youth employed internal and external resources to give a positive purpose to their journeys. External resources are those that provide material and emotional support, such as friends, family and child protection services. Support networks are crucial, including virtual relations and friendships formed during migration (Corona Maioli et al., 2021; Clayton and Gupta, 2019). Pedro, 18, from Honduras, drew a soccer game as representation of resilience (Fig. 3). Jose shares:

“*I have formed different friendships on the road. And good ones, I feel I have left many doors open in Mexico and that makes me happy. Because I know if I come back one day I have where to go*”

Peer support can happen also without forming a friendship or trust bond (Díaz de León, 2022), for example through exchange of food, information or emergency aid. Manuel shares how ‘peers’ helped him when he fell off the train:

M: “*The train hit me and here I am”* [left leg amputated]

S: “*Did someone rescue you?*”

M: “*Yes, my* compañeros [peers]*, my friends* (…) *they put a cloth on my foot and called an ambulance”*

Internal resources, such as hope, faith and goals, seem to translate into resilience by becoming symbolic meanings given to the migration journey and personal circumstances. Peer interactions are important also for collective building of these internal resources, through shared cultural understandings. Specifically, we identified cultural ‘scripts’, or phrases repeated by several participants that implied a common vision of how to face the journey. These ‘scripts’ highlighted the importance of family, faith, God and moving forward no matter what. For example, these are responses of Pedro and Gustavo when asked what they wanted for their future:

Pedro: *“Having my family safe… and ‘salir adelante’* [going forward] *to help them. And that's it… ‘Primero Dios todo salga bien’* [God willing everything will be alright]*”*

Gustavo, 19, from Honduras*: “Well… ‘de aquí p'arriba, seguir adelante y echarle ganas donde toque y de aquí p'arriba’* [from here going up, going forward and work hard wherever it is, and from here going up]*”*

The motivational tone of *salir adelante* implies a forward-looking vision which was evident in another coping mechanism identified among participants: not speaking about things that hurt. At the same time, as William and Violeta share, resilience is built with experience:

William: “*There are many things one has lived, but one always finds a way to get through them”*

Violeta: “*I have gone forward, but it has cost me a lot; it is not easy for me, to have lived all this*”

As shown by Violeta's quote, resilience can overlap with resistance when it implies a high psychological cost. In fact, resistance is a form of functional distress (Southwick et al., 2014; Fraser et al., 1999), and is a salient feature of forced migration experiences when discrimination is experienced, especially whilst looking for a job:

Violeta: *“Why, if I am a human being just like you*? *Why would I give* [a company] *a bad image? There are huge* [LGBTQ+] *communities in Mexico. Why so much discrimination?* (…) *As a migrant, I don't deserve to be treated badly”*

Violeta shows a consciousness of her ‘being’ a migrant and of the unfairness of some of her experiences. Possibly, consciousness of common humanity is a resource as it can improve confidence in oneself, identified as important by many participants, like Estrella:

*“I was afraid, really afraid, but I tried to appear confident, so that if someone came close they would not see me weak, it was like my defence”*

When asked what advice he would give to someone who wanted to migrate, Rosendo said:

*“Hmm.. that he have faith, that he don't lose it, and that he believe in himself”*

**Fig. 4:**
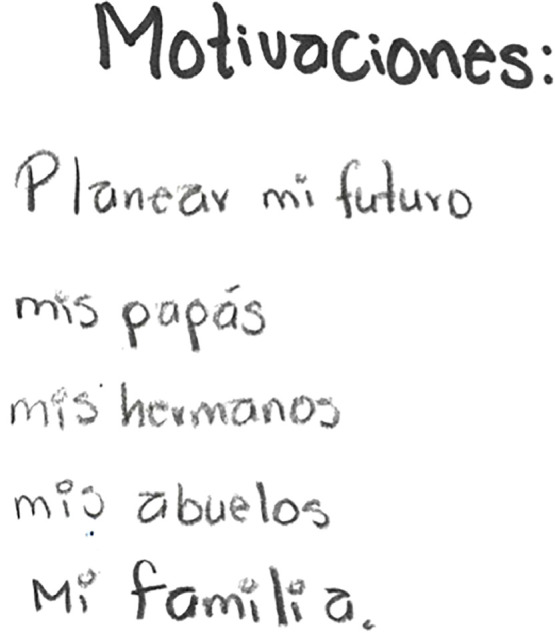
Section of Sara's drawing representing her resilience. Translation: *motivations* [for the journey]: *planning my future, my parents, my brothers, my grandparents, my family*.

Faith in oneself as a resilience mechanism seems to be a result of experience, rather than a shared cultural understanding like faith in God. Faith was also very linked to having a goal and hope to achieve it, which for most participants was helping economically their family or family reunification. Sara listed different family members as her ‘motivation’ (Fig. 4), while Jose shared:

“*It has helped me to think I want to help my family, and thinking about my future* ‘me ha ayudado a salir adelante’ [it has helped me to go forward], *to fight for my dreams. I'm not very religious, but I make an effort and try not to get low*”

## 4 Discussion

Primary contributions of this research are in the fields of transit and unaccompanied child migration, regarding the nuances of highly violent contexts faced by migrant youth and their engagement with this: understood as an effort to overcome the uncertainty and insecurity that occur as a result of structural and interpersonal violence.

Accounts of young people present this context of violence, where gangs sometimes become sites of belonging and survival (Miguel Cruz, 2010). Violence in transit is normalised, pointing to the lack of mention among youth of risks such as trafficking, emphasised by workers. The worrying data void there is about trafficking numbers (Corona Maioli et al., 2021), or other risks migrant children encounter during transit, risks normalising this violence even more. Continuous violence meets what Kara E. Dempsey termed ‘non-linear violence’: a form of violence which disrupts potential for growth (Dempsey, 2020). Nonetheless, despite the important limitations encountered in violent and precarious contexts, where political discrimination hinders safe transit and integration, youth showed how they generated meanings out of their experiences and maintained a goal-oriented projection of themselves – important for identity building. Family had an important role in this: subordination of personal choices over helping the family reflects a salient cultural value termed ‘familism’ in literature (Valdivieso-Mora et al., 2016).

Family and school have been recognised as the first environments where children learn social values (Garelli et al., 2006). However, migration for unaccompanied youth is characterised by either family absence or transnational presence, mitigating somewhat the family's direct influence beyond moral values and responsibility. School is also often interrupted due to migration or the need to work. Thus, the environment that may be most important for these youth is the peer environment. Independence from family – either through the construction of an independent identity when there is no family support, or through taking responsibility *for* the family (Corona-Maioli et al., 2024) – is evidence for the agency that these youth are capable of at a young age, understanding agency as ‘the capacity for intentional behaviour’ (Thompson et al., 2021). Commonly, agency and responsibility are ascribed as an adult feature, which has led some scholars to caution at emphasising migrant youth agency (Chase et al., 2021) - because political discourse is quick to use this against them as proof countering their underage (Corona Maioli et al., 2023). However, not recognising agency is a complete misunderstanding of these youth's journeys, leading them to feel alienated and more prone to escape institutional protection (Lajusticia, 2018), possibly into trafficking networks. Rather, this agency could be leveraged: adolescence is an age when there is much exploration of identity (Garelli et al., 2006) and this can be channelled through work or educational programs. In particular, school can be a place for interaction with peers and learning civic values, rights and duties of social ‘contracts’ like citizenship (Garelli et al., 2006).

We highlight peer support as important also for resilience, emphasising the need to recognise this support network beyond the family as crucial for the wellbeing and personal development of migrant youth. Migration implies ruptures and the challenge for identity to maintain coherence between different times and spaces (Clayton and Gupta, 2019; Timotijevic and Breakwell, 2000; Bromley, 1988): contact with friendships formed during the journey becomes crucial in uniting the dots between an origin and a destination. At the same time, identifying with peers within the ‘migrant identity’ for the purposes of mutual aid and survival – which can foster resilience – may limit identity building due to the social discrimination imposed upon being a ‘migrant’. The ‘migrant identity’ carries a politically charged stigma: for some, like Violeta, this translates into their mere existence becoming resistance and fight.^8^ But it is worth questioning what happens when the body becomes political. Borrowing a term from Achille Mbembe (Mbembe, 2019), ‘embodiment’ of injustice can result in deaths unaccounted for (Schindel, 2020; Arendt, 2009), or in the physical and mental health burden of sustaining an existential fight (Selvarajah et al., 2022).

We cannot speak of identity or resilience without considering the external factors that, in this case, restrict identity building for youth and convert resilience into resistance and survival. Threats to identity and resilience come not from migration itself, to which resilience mechanisms were encountered: such as cultural meanings, goals, learnings. Rather, the conditions in which migration occurs exceed coping abilities and push youth into an abyss of substance use, violence or exploitation. Addressing structural challenges to personal development of migrant youth means recognising our trans-local existence as humans and our shared responsibility towards younger generations. Children and adolescents must be conceptualised as ‘owners’ of their migration processes – rather than adjuncts of or functional to a family unit (Glockner Fagetti and Álvarez Velasco, 2021). Representing migrant youth and defending their best interests can be considered an act of health promotion as defined in the Ottawa Charter (who.int 1986), by promoting environments that foster collective wellbeing.

In summary, key points to be reinforced in services for migrant youth, as well as explored in further research, are: leveraging adolescents’ agency (such as with access to work or educational programs), enhancing peer interactions (including with host population as a form of integration), and expanding a counter-vision of migration as a constitutive part of our societies.

### 4.1 Limitations

Limitations of this study are, first, data derived from one point in time, obscuring the evolution of the lived experiences explored. Fieldwork time was finalised halfway due to Covid-19, limiting documentation of further accounts. Migrant youth outside of shelters, at northern or southern borders, or in more vulnerable situations such as active trafficking, were not included due to logistical and ethical challenges, obscuring their experiences. Finally, more time to build confidence with some participants could have aided deeper responses. Migration as a research field implies high rates of change, with little time to build trust with participants (Greatrick et al., 2022), but documenting lived experiences at a micro-level is mandatory to critically engage with the social and political issues of our times (Glockner Fagetti, 2019).

## 5 Conclusion

This article presented lived experiences of 18 migrant youth interviewed while in transit through Mexico, illustrating violent contexts they flee from and encounter. Focusing on identity building and resilience mechanisms, findings showed that youth maintain a goal-oriented future projection of themselves, they learn from the migration process and ascribe meanings to their journeys. This shows that, through resilience and resistance mechanisms presented, migration can contribute to positive personal development if it occurs in the right conditions. Complementary interviews with 29 professionals described a precarious context of transit through Mexico, which creates significant risks for youth personal development. Ultimately, a vision of common humanity that legitimises expanded health and education services for everyone could benefit younger generations of any nationality, establishing future patterns of health and wellbeing.

## CRediT authorship contribution statement

**Susanna Corona Maioli:** Writing – review & editing, Writing – original draft, Visualization, Validation, Supervision, Software, Resources, Project administration, Methodology, Investigation, Funding acquisition, Formal analysis, Data curation, Conceptualization. **Delan Devakumar:** Writing – review & editing, Validation, Supervision, Data curation, Conceptualization. **Shoshana Berenzon Gorn:** Writing – review & editing, Validation, Supervision, Data curation. **Rochelle A. Burgess:** Writing – review & editing, Validation, Supervision, Methodology, Formal analysis, Data curation, Conceptualization.

## Declaration of competing interest

The authors declare that they have no known competing financial interests or personal relationships that could have appeared to influence the work reported in this paper.

## Appendix Supplementary materials

Image, application 1

## Footnotes

1 According to the General Comment no.6 of the UN Committee on the Rights of the Child, an “unaccompanied minor” is defined as a child or adolescent under the age of 18, who is “separated from both parents and other relatives and is not being cared for by any other adult who, by law or custom, is responsible for doing so”

2 For example, in the development of chronic diseases, metabolic syndrome, depression, anxiety, health harming behaviour, exacerbated by poor access to healthcare and poor occupational safety for many low-wage migrant workers

3 These images are published in: https://www.raceandhealth.org/post/august-2022-migration-and-south-america

4 Human Trafficking is the recruitment, transportation, transfer, harbouring or receipt of people through force, fraud or deception, with the aim of exploiting them for profit

5 In Spanish, ‘falcons’, is the colloquial name used for people who keep watch over who enters and leaves areas controlled by organised crime

6 The “zero tolerance” policy employed by the Trump administration from April to June 2018 separated families by referring adults for prosecution for illegal entry and reclassifying children as unaccompanied. Testimonies of children separated from parents and held in custody of US government: https://www.nytimes.com/2018/07/14/us/migrant-children-shelters.html

7 Migrant Protection Protocols (MPP) is a policy which requires asylum seekers to wait for their claim resolution in Mexico under risky circumstances for a period of 6 months to 2 years

8 Following Achille Mbembe's argument, when racism reaches the extreme point of not allowing life, the mere existence of the racialised body is a form of resistance: being alive is in opposition to a form of lethal racism
