# Supplementary material for: Growing up in transit. Personal development and resistance of migrant adolescents travelling through Mexico unaccompanied
Source: J Migr Health. 2024 Jul 14;10:100245. doi: 10.1016/j.jmh.2024.100245 (PMC11915520; doi:10.1016/j.jmh.2024.100245)
Supplement: Supplementary file 1 [file mmc1.docx]

**Growing up in transit.**

**Personal development and resistance of migrant adolescents travelling through Mexico unaccompanied**

**Annex**

**Authors:** Susanna Corona Maioli [1], Delan Devakumar [1], Shoshana Berenzon Gorn [2], Rochelle Anne Burgess [1]

Table of Contents

[Topic guide for interviews with migrant youth 2](#_Toc159590324)

[Topic guide for interviews with professionals 3](#_Toc159590325)

[Thematic categories of migrant youth 4](#_Toc159590326)

[Thematic categories of workers 5](#_Toc159590327)

# **Topic guide for interviews with migrant youth**

| **Temas / *Topics*** | **Posibles preguntas / *Possible questions*** |
| --- | --- |
| Introducción / *Introduction* | ¿De dónde vienes? / *Where do you come from?*  ¿Por qué viajas? / *Why do you travel?*  [primary reason, secondary reasons?]   ¿Cuánto tiempo llevas viajando o en México?  / *How long have you been travelling and/or in Mexico?*   ¿Cuánto tiempo llevas/te quedarás en este Centro?  / *How long have you been in this shelter?* |
| Experiencia del tránsito /  *Transit experience* | ¿Cómo vives/has vivido el cambio constante de lugar?  / *How do you live/have you lived the constant change in place?*   ¿Has intentado quedarte en un lugar más tiempo?  / *Have you tried to stay in a place for longer?*     ¿Cómo ha sido el camino hasta ahora?  / *How has the journey been until now?* |
| Identidad / *Identity* | ¿Te identificas con algún grupo?  / *Do you identify with any group?*   ¿Desde cuándo te identificas con este grupo?  / *Since when?*   ¿Crees que ha cambiado tu manera de pensar en ti mismo desde que empezaste tu viaje?  / *Do you think the way you think about yourself has changed since you started your journey?*   ¿Por qué crees que ha cambiado?  */ Why?*   ¿Cuánto influyen los demás en tu manera de verte a ti mismo?  / *How much do others matter in your way to see yourself?* |
| Resiliencia / *Resilience* | ¿Qué te ha ayudado a seguir adelante?  / *What has helped you move forward?*  ¿Tienes algún objeto simbólico que te da fuerza?  / *Do you have a symbolic object that gives you strength?*  [Conocimiento] ¿Sabías algo de cómo iba a ser tu viaje? ¿Cómo te informaste / quién te informó?  / *Did you know anything about how the journey would be? How did you inform yourself / who informed you?*  [Capital social] ¿Has formado amistades en el camino? ¿Estás en contacto con tu familia? ¿Tienes quién te reciba en tu destino?  / *Have you formed friendships on the way? Are you in contact with your family? Do you have someone to receive you in your destination?*  [Aprendizaje] ¿Te sientes más seguro ahora que has viajado un poco? ¿Por qué?  / *Do you feel more confident now that you have journeyed some time? Why?* |
| Apoyo / *Support* | ¿Qué tipo de apoyo consideras más importante para ti? (material, solidaridad e integración, mejor información legal, etc)  / *Which type of support is more important to you? Why?*  ¿Conoces tus derechos? ¿Sabes que se pueden hacer quejas ante la CNDH? ¿Sabes sobre la posibilidad de pedir refugio en México?  / *Do you know your rights? Do you know you can file a complaint with CNDH? Do you know about the possibility of seeking refuge in Mexico?* |

# **Topic guide for interviews with professionals**

| **Temas / *Topics*** | **Posibles preguntas / *Possible questions*** |
| --- | --- |
| Introducción / *Introduction* | ¿Trabaja directa o indirectamente con menores y/o jóvenes migrantes?  *Do you work directly with unaccompanied children?*  ¿Qué tipo de trabajo hace con/para menores y/o jóvenes migrantes? ¿Desde cuándo?  *What type of work do you do with unaccompanied children? Since when?* |
| Idoneidad de servicios / *Service competence* | ¿En que medida cree que los servicios que atienden a menores y/o jóvenes migrantes presentes en México son adecuados a la necesidad?  / *How competent do you think services that care for unaccompanied minors and/or youth are in Mexico, compared to the need?*  ¿Cómo cree que es el contexto que enfrentan los menores no acompañados al llegar a México?  / *How do you think is the context that UAMs and/or youth face when they reach Mexico?*  ¿Cree que las leyes actuales -cómo la LGDNNA- responden a la necesidad?  / *Do you think current legislation – such as LGDNNA – responds to these needs?* |
| Falta de servicios / *Lack of services* | ¿Qué cree que se podría mejorar en los servicios presentes en México que atienden a menores y/o jóvenes migrantes?  / *What do you think can be improved in the services for unaccompanied minors and/or youth in Mexico?*  ¿Cree que las leyes presentes -cómo la LGDNNA- son implementadas? ¿Por qué?  / *Do you think current legislation – such as LGDNNA – is implemented? Why?* |

# **Thematic categories of migrant youth**

| **Thematic category** | **Themes** | **Sub-themes** | **Codes** |
| --- | --- | --- | --- |
| **Embedded violence in the environment** | - Peer culture – impunity, corruption - Violence as survival | - Domestic violence - Sexual violence - Gang violence | Extortion; threats; corruption; impunity; gangs in the family |
| **Lack of freedom to develop and choose** | - Change as constant adaptation and less stable references - Waiting/delay - Psychological cost | - Change of home - Change of plans - Personal change - Transit - Inappropriate housing - Psychological fight - Difficulties and unmet needs | Escapes from institutionalised accommodation; voluntary return; deportation; staying in Mexico; adjusting to differences; train tracks; detention; interaction with National Institute for Migration (INM) or Guardia Nacional; activities to pass the time in institutionalised accommodation; discrimination; fear; mistrust; witnessing; mutilation |
| **The family cultural ideal and the family reality** | - Maltreatment by close family members - Peer culture – responsibility to help the family and family as a goal to seek | - Negligence (family presence but no support for development) - Lack of family presence or family resources – also leading to lack of support for development - Loss of close family members (by migration or death) | Lack of life plan; lack of study and formal education; leaving school; living without parents; remembering; sadness; craving; homesickness; mother |
| ***Salir adelante* [going forward] as a narrative of hope, personal development and goal achievement** | - Internal resources - External resources | - Agency/decision making - Faith - Goals - Strength - Shelter support - Personal support | Learning how to survive on their own (accumulated transit/travel experience; independence; figuring out the way; planning the trip; *no contar* (not telling); joining other people; letting go; God; family reunification; work; United States; *superarse* (personal development); buying a house; hope; courage; humanity; peer support; temporary/virtual friendships; acceptance for who they are (eg LGBTQ) |

# **Thematic categories of workers**

| **Thematic category** | **Themes** | **Sub-themes** | **Codes** |
| --- | --- | --- | --- |
| ***Necesidad sin abasto* [unmet need on behalf of authorities and child protection]** | - Lack of structural resources - Lack of human resources | - Lack of capacity - Lack of continuity - Lack of coordination - Pandemic impact - *Acompañamiento* [appropriate follow up] - *Capacitación* [training of workers] - *Modelos de atención* [care guidelines] | Little time (spent in shelters by migrant youth); (teaching) perseverance and determination; (teaching) discipline; first detection; first reception; longer term care; integration/independent life; age of majority |
| ***Acompañamiento* [representativeness]** | - Worker skills and capabilities - Worker knowledge | - Adaptation - Responsibility - Empathy - Listening ability - Recognition - (Knowledge of) migration context - (Knowledge of) best interests of the child - (Knowledge of) interview techniques - (Knowledge of) advocacy techniques | Gratification; legal identity (articles 7, 8 UNCRC); family reunification (article 10 UNCRC); education (article 28 UNCRC); integration (articles 22, 39 UNCRC); health and mental health (article 24 UNCRC) |
| **Politics and vision** | - Institutional/   official response   - Public/civil society response | - LGDNNA - Procuraduría/DIF [Child Protection Authority/Child Welfare] - Regularisation - Visibility | Advocacy; invisibility |
| **Vulnerabilities of migrant youth from worker perspective** | - Trafficking - Substance abuse - Exploitation |  |  |
